# Safety and clinical performance of intra-articular high-purity sodium hyaluronate injection in patients with knee osteoarthritis: a 28-week post-market clinical follow-up study

**DOI:** 10.1007/s10067-025-07891-1

**Published:** 2025-12-24

**Authors:** Cheng-Fong Chen, Ching-Ting Chen, Yung-Hsiang Lin, Chi-Fu Chiang, Yi-Wen Mao

**Affiliations:** 1 Division of Joint Reconstruction, Department of Orthopedics and Traumatology, Taipei Veterans General Hospital, Taipei, Taiwan; 2 Department of Surgery, School of Medicine, National Yang Ming Chiao Tung University, Taipei, Taiwan; 3 Maxigen Biotech Inc., Taoyuan, Taiwan; 4 Research & Design Center, TCI Co., Ltd., Taipei, Taiwan

**Keywords:** Hyaluronic acid, Intra-articular injection, Knee osteoarthritis, Pain management, Post-market clinical follow-up

## Abstract

**Objectives:**

Intra-articular hyaluronic acid (HA) is a widely used treatment for knee osteoarthritis (OA), offering localized symptomatic relief with minimal systemic exposure. This post-market clinical follow-up (PMCF) study aimed to evaluate the safety and clinical performance of a high-purity sodium hyaluronate formulation in a real-world population with symptomatic knee OA.

**Methods:**

In this prospective, single-arm, open-label post-market follow-up study, 65 patients with radiographically confirmed knee OA received three weekly intra-articular injections of a high-purity sodium hyaluronate formulation (1.5%, 15 mg/mL). Patients were followed through Week 28. The primary outcome was safety, assessed by adverse event (AE) monitoring and vital signs. Secondary assessments included radiographic Kellgren–Lawrence (KL) grading as part of structural safety monitoring, resting knee pain, and patient satisfaction, both assessed using 100-mm visual analog scales (VAS).

**Results:**

No serious treatment-related adverse events (AEs) were reported. Mild, transient joint pain and swelling occurred in two participants (3.1%) and resolved without intervention. VAS pain scores improved significantly from baseline (41.5 ± 23.1 mm) to Week 6 (17.6 ± 19.6 mm, *p* < 0.001) and Week 28 (12.7 ± 17.3 mm, *p* < 0.001). Median KL grade remained stable (median 2, IQR 2–3) over the study period, with no acute or unexpected structural changes. At Week 28, 88.1% of participants reported being satisfied or very satisfied.

**Conclusions:**

Intra-articular injection of high-purity sodium hyaluronate was well tolerated and associated with significant pain reduction and high patient satisfaction, with no radiographic signs of acute or unexpected structural deterioration. These findings support the use of this high-purity HA formulation as a safe and effective option for symptomatic management of knee OA in real-world clinical practice.

**Trial registration:**

This study was registered at ClinicalTrials.gov (Identifier: NCT05881317; registered May 31, 2023).

**Table:** 

**Key Points** • *High-purity intra-articular sodium hyaluronate was well tolerated, with only mild, self-resolving adverse events.* • *Significant and sustained reduction in knee pain was observed through 28 weeks of follow-up.* • *Radiographic Kellgren–Lawrence grades remained stable at both short-term and mid-term safety evaluations.* • *Most patients reported high satisfaction, supporting the formulation as a safe and effective real-world option for symptomatic knee osteoarthritis.*

**Supplementary information:**

The online version contains supplementary material available at https://doi.org/10.1007/s10067-025-07891-1.

## Introduction

Osteoarthritis (OA) is a prevalent, degenerative joint disease characterized by progressive cartilage deterioration, subchondral bone remodeling, osteophyte formation, and synovial inflammation, ultimately leading to chronic pain, joint stiffness, and functional limitations. It is estimated that symptomatic knee OA affects approximately 10% of men and 13% of women aged 60 years or older [1]. As a major global health burden, OA contributes significantly to disability and places increasing demands on healthcare systems, trends that are exacerbated by population aging and rising obesity rates [2].

Management of knee OA typically follows a stepwise approach—including patient education, weight control, physical therapy, and pharmacologic interventions such as nonsteroidal anti-inflammatory drugs (NSAIDs) or acetaminophen—consistent with ESCEO guidance on non-surgical management of knee OA [3]. However, long-term oral use of systemic agents is limited by an elevated risk of gastrointestinal, cardiovascular, and renal adverse effects, particularly among elderly patients [4]. Consequently, intra-articular (IA) therapies have gained popularity as localized alternatives that provide symptom relief with minimal systemic exposure. Among these, IA hyaluronic acid (HA) injections are widely adopted for viscosupplementation in patients who remain symptomatic despite conservative treatment. Recent reviews support the efficacy and safety of HA injections in reducing pain and improving function in knee OA, especially in populations with contraindications to systemic therapies [5, 6].

HA is a naturally occurring glycosaminoglycan present in synovial fluid, where it plays a crucial role in maintaining joint homeostasis through its viscoelastic properties that support lubrication, shock absorption, and load distribution [7]. In OA, both the concentration and molecular weight of endogenous HA decline, impairing these protective biomechanical properties and exacerbating inflammation within the joint space [8]. Intra-articular administration of exogenous HA aims to replenish these rheological deficits and may exert biological effects, such as downregulating pro‑inflammatory cytokines (e.g., IL‑1β, TNF‑α) and inhibiting matrix metalloproteinases (MMPs), thus reducing pain and potentially slowing cartilage degradation [9, 10]. Despite these multifaceted mechanisms, individual responses to HA treatment remain inconsistent across clinical studies, underscoring the need for further investigation into determinants of efficacy.

While multiple randomized controlled trials (RCTs) and meta-analyses support the safety and efficacy of intra-articular hyaluronic acid (IA-HA), inconsistencies remain among clinical guidelines regarding its routine use. These differences are partly attributed to variations in patient characteristics, HA formulation properties, and study methodologies [11]. Therefore, real-world post-marketing studies are essential to evaluate HA performance in broader clinical populations that are often underrepresented in RCTs.

This prospective, open-label study aimed to evaluate the safety and clinical outcomes of a cross-linked, high-purity sodium hyaluronate formulation over a 28-week follow-up period in real-world patients with radiographically confirmed knee OA. Eligible patients either failed to achieve adequate relief from conservative therapy or could not tolerate systemic medications. The primary endpoint was the incidence of adverse events following intra-articular administration. Secondary outcomes included resting pain (visual analog scale, VAS) and patient satisfaction (VAS-based rating). Radiographic Kellgren–Lawrence (KL) grading was performed at baseline, Week 6, and Week 28 as part of structural safety monitoring to ensure that no acute or unexpected morphological deterioration occurred following intra-articular injection. The findings from this study contribute to the real-world evidence supporting HA therapy as a safe and symptomatically beneficial intervention, especially in elderly patients and those unsuitable for systemic pharmacologic options.

## Materials and methods

### Study design and ethical considerations

This prospective, single-arm, open-label clinical trial was conducted at Taipei Veterans General Hospital, Taiwan, with patient enrollment from August 2, 2021, to June 2, 2022, and study completion on November 21, 2022. No randomisation, allocation concealment, or control group was employed in the study, given its single-arm post-market design. The aim was to evaluate the safety and clinical performance of a cross-linked, high-purity sodium hyaluronate formulation (ArtiAid® Plus) administered via intra-articular injection in patients with knee osteoarthritis (OA). The trial was designed in accordance with the Declaration of Helsinki, Good Clinical Practice (ICH E6 R2), and applicable local regulations. The study protocol was approved by the Institutional Review Board of Taipei Veterans General Hospital (IRB No. 2021–02-001A#1), and written informed consent was obtained from all participants prior to enrollment. This trial was conducted as a post-market clinical follow-up (PMCF) study in accordance with the European Medical Device Regulation (MDR) and Taiwan’s regulatory requirements for Class III medical devices. Although the trial registration (ClinicalTrials.gov Identifier: NCT05881317) occurred after study completion, the study was conducted entirely in accordance with the IRB-approved protocol and applicable ethical standards.

#### Participants

Patients were recruited from orthopedic outpatient services and screened based on clinical symptoms, physical examination, and radiographic confirmation of knee OA. Inclusion criteria were: as follows (1) age > 40 years; (2) radiographic diagnosis of Kellgren-Lawrence (KL) grade II–III knee OA; (3) inadequate symptom control despite conservative, non-pharmacologic interventions; (4) negative pregnancy test in women of childbearing potential; and (5) clinically stable condition, as determined by the investigator.

Key exclusion criteria included (1) hypersensitivity to hyaluronate or its excipients; (2) active local infection or skin disease at the injection site; (3) pregnancy or lactation; (4) history of alcohol or substance abuse; (5) participation in other clinical trials within 3 months prior to enrollment; and (6) recent overseas travel (within 3 months before screening).

#### Intervention

All eligible participants received three weekly intra-articular injections of a high-purity sodium hyaluronate formulation (1.5%, 15 mg/mL; 2 mL per injection; lot AAP-2110) administered at Visit 2, Visit 3, and Visit 4. The investigational product (ArtiAid® Plus, Maxigen Biotech Inc., Taiwan) was provided in pre-filled syringes containing sterile, pyrogen-free sodium hyaluronate in buffered physiological saline. Injections were performed by trained clinicians using standard aseptic techniques and anatomical guidance. No corticosteroids or local anesthetics were co-administered. Participants were monitored through Visit 7 (Week 28). Concomitant use of systemic analgesics or other intra-articular treatments was not permitted during the study period.

### Outcome measures

#### Primary outcome

The primary endpoint was safety, evaluated by monitoring adverse events (AEs) and serious adverse events (SAEs) from baseline through Week 28. AEs were assessed by investigators for severity (per National Cancer Institute Common Terminology Criteria for Adverse Events, version 4.0 [NCI CTCAE v4.0]), duration, required interventions, outcomes, and their relationship to the study product (categorized as definite, probable, possible, unlikely, or unrelated). Vital signs—including blood pressure, pulse rate, and tympanic temperature—were recorded at each visit to monitor general safety.

#### Secondary outcomes

The secondary objectives were to assess symptomatic pain relief and patient-reported satisfaction following treatment. Radiographic Kellgren–Lawrence (KL) grading was also performed at baseline (Visit 1), Week 6 (Visit 5), and Week 28 (Visit 7) solely as part of structural safety monitoring. The interim radiographic evaluation at Week 6 was included as an early safety check to verify that no acute morphological deterioration or unexpected structural abnormalities occurred following intra-articular injection. This imaging served as a short-term checkpoint for joint stability prior to the final Week-28 evaluation. Pain intensity was measured at the same timepoints using a 100-mm visual analog scale (VAS) for resting knee pain. Treatment satisfaction was assessed at Visit 5 and Visit 7 using a similar 100-mm VAS, with higher scores indicating greater satisfaction. KL grading was performed by trained assessors blinded to visit sequence, and VAS scores were recorded separately for each treated knee. Pain- and satisfaction-related secondary outcomes were selected to capture patient-reported treatment responses. All primary and secondary outcome measures were pre-specified in the IRB-approved protocol prior to study initiation, and no changes were made after the trial commenced.

#### Statistical analysis

Sample size was calculated based on Charan and Biswas’ method for observational studies with binary outcomes, assuming a type I error of 5%, a desired precision of ± 12%, and an expected dropout rate of 15%. The estimated incidence of adverse events was < 10%, based on prior post-marketing safety data, yielding a target enrollment of 65 participants [12].

Efficacy analyses were conducted in the per-protocol (PP) population, defined as participants who completed all scheduled visits and assessments. Safety analyses were based on the intention-to-treat (ITT) population, which included all participants who received at least one injection.

Continuous variables were summarized as means and standard deviations, and categorical variables as frequencies and percentages. For continuous variables, paired comparisons across timepoints were analyzed using Student’s *t*-tests. Repeated-measures analysis of variance (ANOVA) was employed to evaluate within-subject changes over time. Ordinal data were analyzed using nonparametric methods, with the Wilcoxon signed-rank test applied for paired comparisons and the Friedman test used for repeated measures. A *p*-value < 0.05 was considered statistically significant. Statistical analyses were performed using SAS® software, version 9.4 (SAS Institute Inc., Cary, NC, USA). No imputation was performed for missing values. No interim analyses or early stopping rules were applied during the study.

## Results

### Participant flow and baseline characteristics

**Fig. 1:**
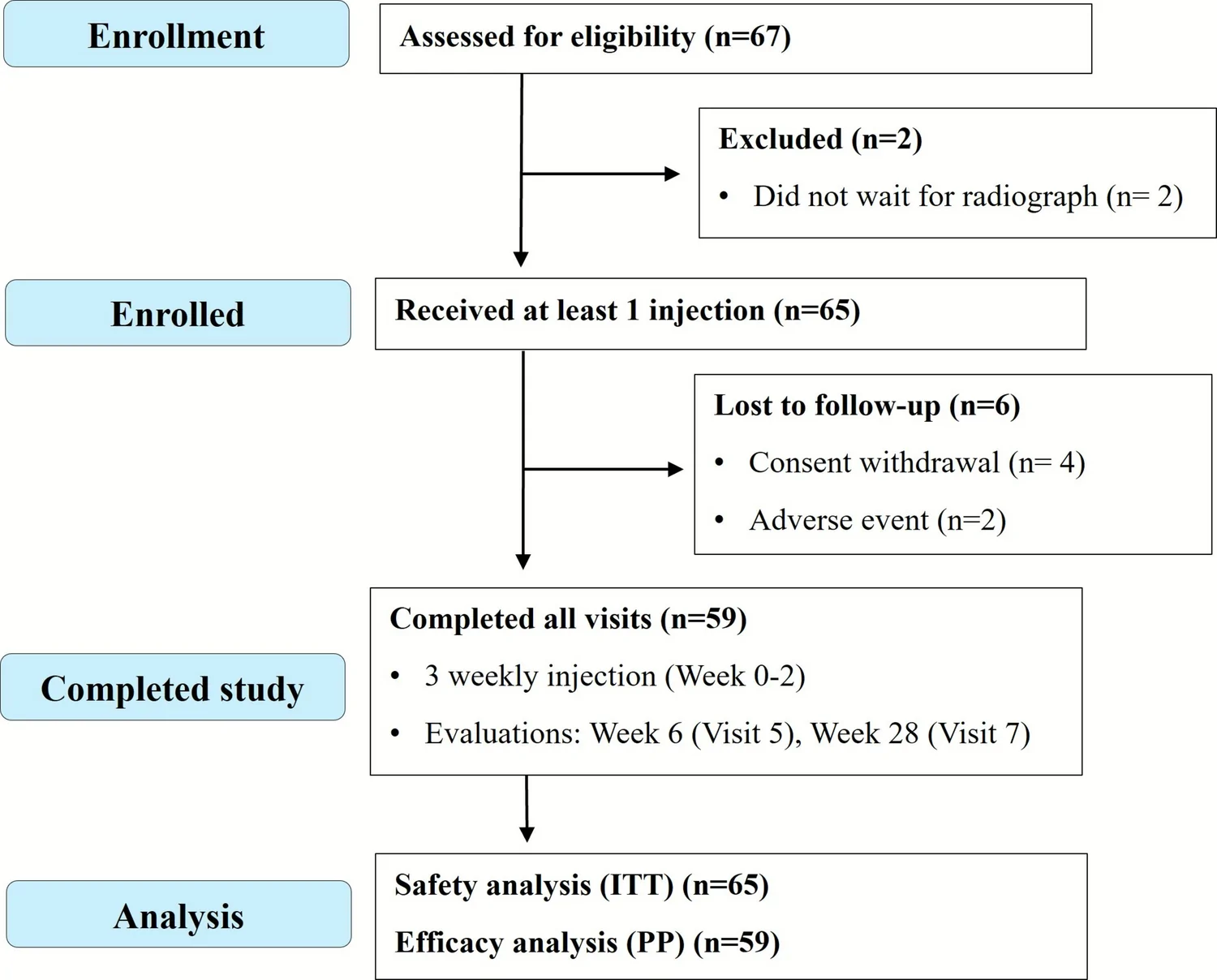
Flow diagram of participant screening, enrollment, treatment, and analysis. A total of 67 patients were assessed for eligibility; two were excluded due to missing radiographic data. Sixty-five patients received at least one intra-articular injection and were included in the safety population (intention-to-treat, ITT). During the 28-week follow-up period, six participants discontinued (three due to consent withdrawal and three due to adverse events), resulting in 59 participants completing all scheduled visits and forming the efficacy population (per-protocol, PP). Injections were administered between Visit 2 and Visit 4, with follow-up assessments at Visit 5 (Week 6) and Visit 7 (Week 28)

To characterize the study population and document subject disposition, participant flow and baseline data were summarized. A total of 67 patients were screened, of whom 65 received at least one intra-articular injection and were included in the intention-to-treat (ITT) safety analysis. Six participants discontinued the study during the 28-week follow-up (three due to consent withdrawal and three due to adverse events), resulting in 59 participants who completed all scheduled visits and were included in the per-protocol (PP) efficacy analysis. The study was completed as planned without early termination. The participant flow is illustrated in Fig. 1.

The mean age of the cohort was 62.7 ± 9.0 years, and the mean body mass index (BMI) was 25.1 ± 4.4 kg/m^2^. Most participants were female (81.5%). At baseline, 63.1% had KL grade II osteoarthritis and 36.9% had KL grade III. Bilateral knee involvement was present in 73.9%, with 16.9% and 9.2% involving only the right or left knee, respectively. The average baseline resting pain score was 41.5 ± 23.1 mm on a 100-mm visual analog scale (VAS). Detailed baseline data are presented in Table 1.

**Table 1:** Baseline demographic and clinical characteristics of enrolled participants

Characteristics	Sodium hyaluronate injection
Demographic characteristics	
Age, mean ± SD, yrs	62.7 ± 9.04
Sex, *n* (%)	
Male	12 (18.5%)
Female	53 (81.5%)
Weight, mean ± SD, kg	63.4 ± 12.43
Height, mean ± SD, cm	158.6 ± 7.30
BMI, mean ± SD, kg/m^2^	25.1 ± 4.41
OA disease characteristics	
Kellgren-Lawrence grade	
Grade II (mild), *n* (%)	41 (63.08%)
Grade III (moderate), *n* (%)	24 (36.92%)
Bilateral knee, *n* (%)	48 (73.85%)
Right knee, *n* (%)	11 (16.92%)
Left knee, *n* (%)	6 (9.23%)
Clinical evaluation	
Pain, mean ± SD, mm	41.53 ± 23.08

Values are expressed as mean ± standard deviation (SD) for continuous variables and as number (percentage) for categorical variables. All treated knees were included in the descriptive analysis of Kellgren–Lawrence (KL) grade and resting VAS pain. KL grades were recorded per treated knee, including bilateral cases. VAS scores represent the mean resting pain per knee at baseline

### Radiographic structural safety assessment (KL Grade)

**Fig. 2:**
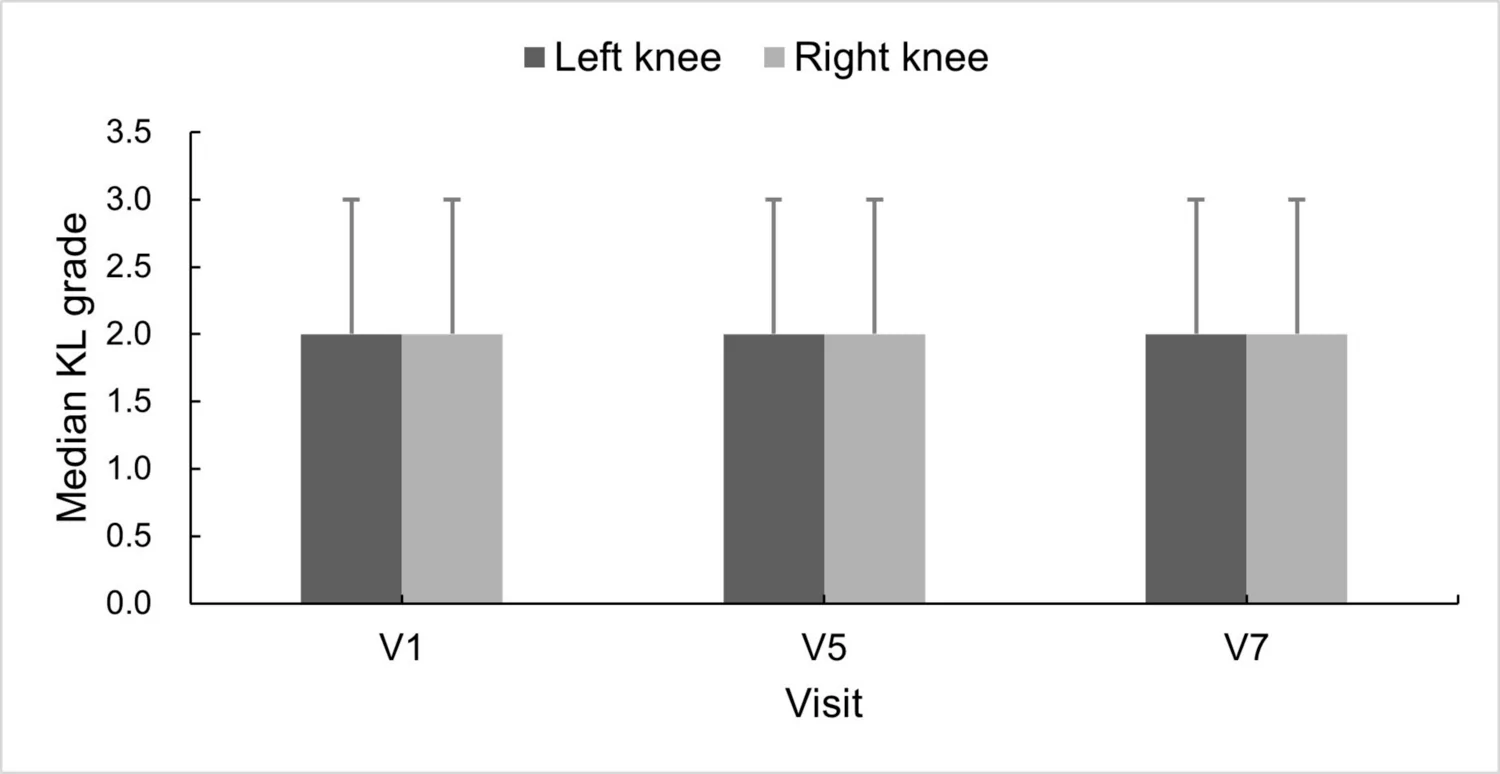
Median Kellgren–Lawrence (KL) grades of left and right knees at baseline (Visit 1), Week 6 (Visit 5), and Week 28 (Visit 7). Radiographic KL grades were assessed separately for each treated knee. Bars indicate median with interquartile range (IQR). No statistically significant differences were observed across timepoints (Friedman test,* p* = 0.985; Wilcoxon signed-rank test, all* p* = 0.317)

To evaluate potential changes in joint structure following treatment, Kellgren–Lawrence (KL) grades were assessed at baseline, Week 6, and Week 28. Among the per-protocol population (*n* = 59), median KL grades remained stable at 2 (interquartile range [IQR] 2–3) for both left and right knees across all timepoints. Wilcoxon signed-rank tests showed no significant changes from baseline to Week 6 or Week 28 (all *p* = 0.317), and the Friedman test across all treated knees also indicated no significant effect of time (*p* = 0.985) (Table 2, Fig. 2). These findings indicate a stable radiographic appearance, with no acute or unexpected structural changes observed over the 28-week follow-up.

**Table 2:** Median Kellgren–Lawrence grades and paired comparisons over time for left and right knees

Timepoint	Knee	*N*	KL grade (median, IQR)	Wilcoxon signed-rank test vs. baseline (*p*-value)
V1	Left	54	2, 2–3	—
V5		50	2, 2–3	0.317
V7		50	2, 2–3	0.317
V1	Right	59	2, 2–3	—
V5		55	2, 2–3	0.317
V7		54	2, 2–3	0.317

Radiographic KL grades were assessed for each knee at baseline (Visit 1), Week 6 (Visit 5), and Week 28 (Visit 7). The Wilcoxon signed-rank test was used to compare each follow-up timepoint with baseline. No statistically significant differences were observed. The Friedman test for all treated knees also showed no significant effect of time (*p* = 0.985). The corresponding trend is shown in Fig. 2

### Resting pain reduction (VAS Scores)

**Fig. 3:**
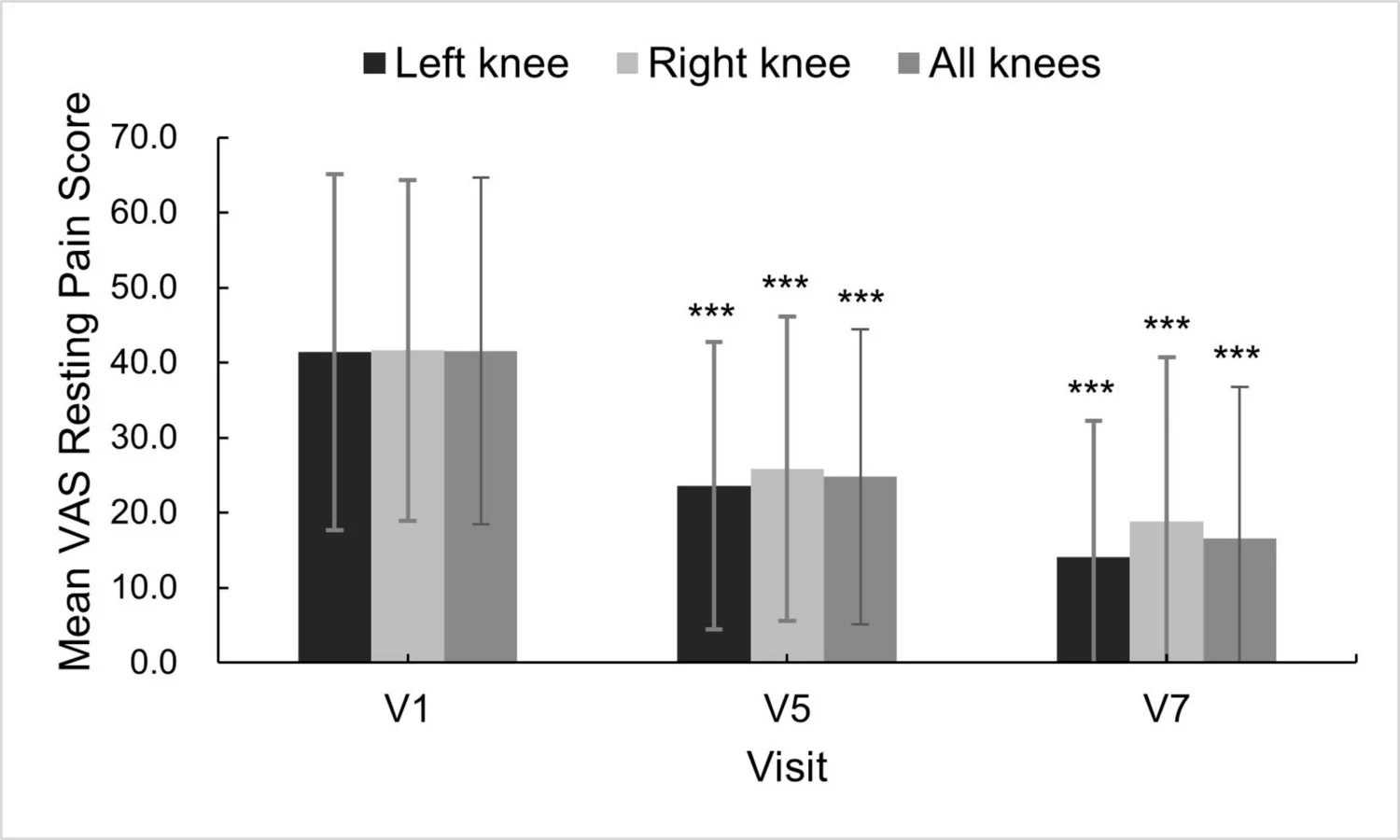
Changes in mean visual analog scale (VAS) scores for resting knee pain over time. Pain was assessed at baseline (Visit 1), Week 6 (Visit 5), and Week 28 (Visit 7) using a 100-mm VAS. Bars indicate group mean ± SD for left, right, and all treated knees. Significant reductions in pain were observed at both follow-up visits compared to baseline for all groups (***p < 0.001, paired *t*-test)

To assess symptomatic improvement, resting knee pain was evaluated using a 100-mm visual analog scale (VAS). In the per-protocol population, the mean VAS score for all treated knees significantly decreased from baseline (41.5 ± 23.1 mm) to Week 6 (24.8 ± 19.7 mm, *p* < 0.001), and further declined to 16.6 ± 20.2 mm at Week 28 (*p* < 0.001) (Table 3, Fig. 3). When analyzed separately, both left and right knees showed consistent patterns of improvement. For left knees, mean VAS scores decreased from 41.4 ± 23.7 mm to 23.6 ± 19.1 mm at Week 6 and 14.1 ± 18.1 mm at Week 28. For right knees, scores declined from 41.6 ± 22.7 mm to 25.9 ± 20.3 mm and 18.9 ± 21.8 mm, respectively (all *p* < 0.001). These results demonstrate a rapid and sustained reduction in resting knee pain following intra-articular treatment, supporting meaningful symptomatic relief over the 28-week observation period.

**Table 3:** Changes in visual analog scale (VAS) resting pain scores over time for left, right, and all treated knees

Timepoint	Knee	*N*	VAS (mean ± SD)	Paired *t*-test vs. baseline (*p*-value)
V1	Left	54	41.41 ± 23.72	–
V5		50	23.58 ± 19.12	< 0.001
V7		50	14.10 ± 18.11	< 0.001
V1	Right	59	41.64 ± 22.69	–
V5		55	25.87 ± 20.28	< 0.001
V7		54	18.89 ± 21.78	< 0.001
V1	All knees	113	41.53 ± 23.08	–
V5		105	24.78 ± 19.68	< 0.001
V7		104	16.59 ± 20.15	< 0.001

VAS scores for resting pain were assessed using a 100-mm visual analog scale at baseline (Visit 1), Week 6 (Visit 5), and Week 28 (Visit 7). Data are presented as mean ± SD. Paired *t*-tests comparing each follow-up timepoint with baseline showed statistically significant reductions in pain for all groups (*p* < 0.001)

### Patient-reported satisfaction

**Fig. 4:**
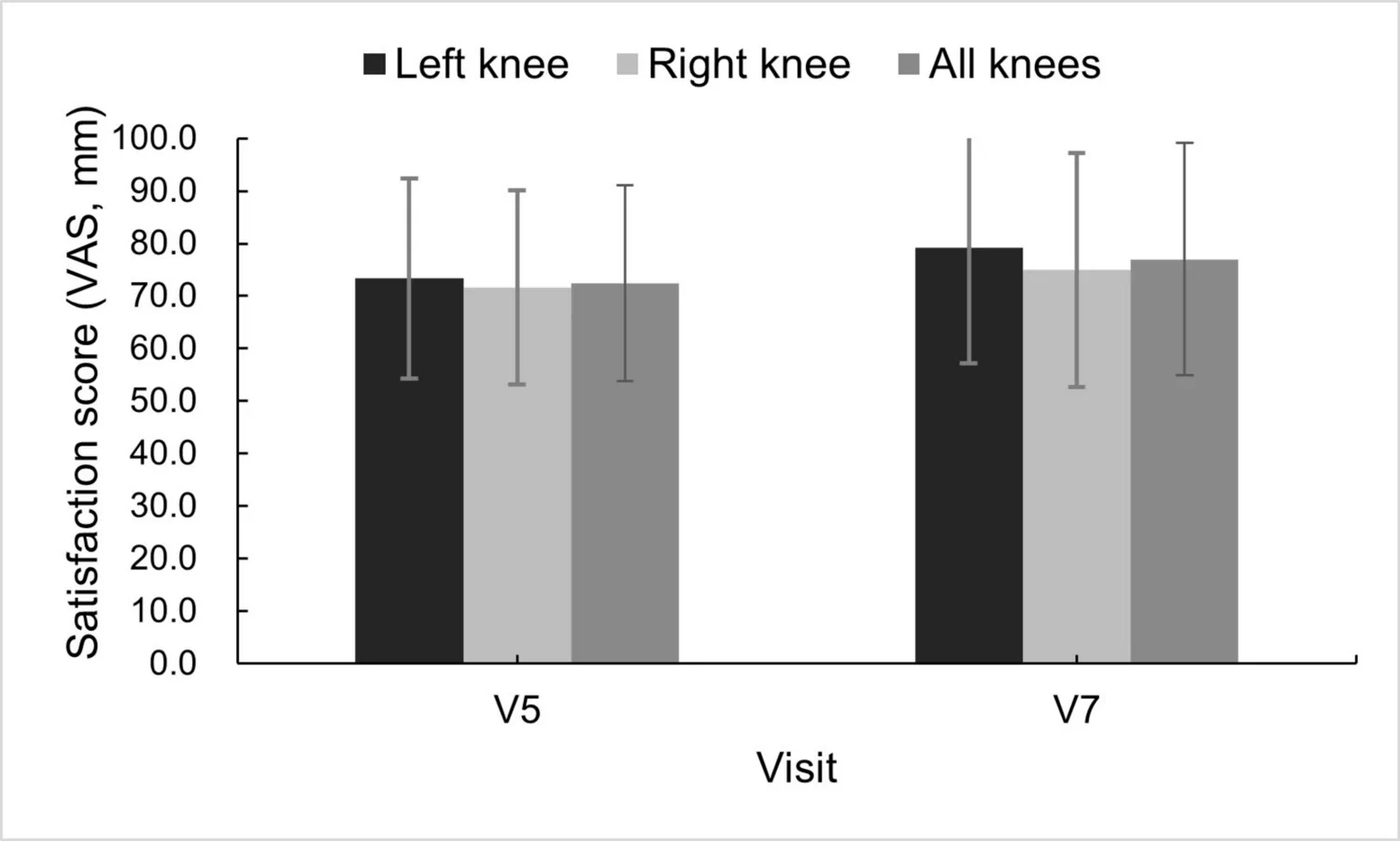
Patient satisfaction scores at Week 6 (Visit 5) and Week 28 (Visit 7) for left, right, and all treated knees. Satisfaction scores were assessed using a 100 mm visual analog scale (VAS) at Week 6 (Visit 5) and Week 28 (Visit 7). Bars represent group mean ± standard deviation (SD) for the left, right, and all treated knees. A slight numerical increase in satisfaction was observed at Week 28; however, no statistically significant differences were found between timepoints (*p* > 0.1 for all comparisons, paired *t*-test)

To evaluate patient-perceived treatment benefit, satisfaction was evaluated using a 100-mm VAS at Weeks 6 and 28. The mean score increased from 72.4 ± 18.6 mm to 77.0 ± 22.1 mm, though the change was not statistically significant (*p* = 0.110) (Table 4, Fig. 4). Satisfaction scores for both knees followed a similar trend.

**Table 4:** Changes in patient satisfaction scores at Week 6 and Week 28 for left, right, and all treated knees

Timepoint	Knee	*N*	Satisfaction (mean ± SD)	Paired *t*-test (*p*-value)
V5	Left	50	73.32 ± 19.02	–
V7		50	79.16 ± 22.03	0.159
V5	Right	55	71.62 ± 18.43	–
V7		54	74.93 ± 22.19	0.399
V5	All knees	105	72.43 ± 18.64	–
V7		104	76.96 ± 22.11	0.110

Patient satisfaction was assessed using a 100 mm visual analog scale at Week 6 (Visit 5) and Week 28 (Visit 7). Data are presented as mean ± standard deviation (SD). Paired *t*-tests were performed to compare scores between the two visits. No statistically significant differences were observed

In addition to the continuous VAS scale, categorical satisfaction ratings were collected at the final visit using a structured questionnaire. Among the 59 participants who completed all scheduled visits, 88.1% reported being satisfied or very satisfied with the treatment, while only 3.4% expressed dissatisfaction. These findings suggest a generally favorable perception of treatment effectiveness among study participants.

### Safety evaluation

To monitor tolerability, adverse events (AEs) and serious adverse events (SAEs) were recorded throughout the study. Among the 65 ITT participants, 17 (26.2%) experienced a total of 37 AEs. Only two AEs (joint swelling and joint pain) were considered related to the investigational product, both mild and self-resolving. No systemic reactions or treatment-related SAEs occurred.

Three SAEs were reported overall; none were attributed to the study treatment. Two of these SAEs led to study withdrawal. No deaths or life-threatening events occurred. Vital signs remained within normal ranges during the study period. A summary of AEs is provided in Table 5, supporting a favorable safety profile for intra-articular administration of this high-purity sodium hyaluronate formulation. No unexpected adverse events or harms were observed during the study.

**Table 5:** Summary of adverse events

Category	Number of events	Number of subjects	% of subjects (*N* = 65)
Total adverse events (AEs)	37	17	26.2%
AEs related to treatment	2	2	3.1%
- Injection site swelling	1	1	1.5%
- Injection site pain	1	1	1.5%
Serious adverse events (SAEs)	3	3	4.6%
- Related to treatment	0	0	0%
Withdrawals due to AE	2	2	3.1%
Deaths	0	0	0%

Adverse events (AEs) were coded using MedDRA version 25.1 and analyzed in the safety population (*N* = 65). Three serious adverse events (SAEs) were reported, two of which led to study discontinuation. None of the SAEs were considered related to the study treatment. All treatment-related AEs were mild in severity

## Discussion

This prospective post-market study evaluated the safety and clinical performance of a cross-linked, high-purity sodium hyaluronate injection in patients with radiographically confirmed knee osteoarthritis (OA) over a 28-week period. The findings demonstrate that the intervention was well tolerated and led to sustained improvements in resting pain and patient satisfaction, with a stable radiographic appearance on safety evaluation, and no acute structural changes observed.

The observed reduction in resting pain, as measured by the visual analog scale (VAS), was both statistically and clinically meaningful. Pain scores declined rapidly by Week 6 and continued to improve through Week 28, aligning with prior research demonstrating the analgesic effects of intra-articular hyaluronic acid (HA), particularly in patients with mild-to-moderate OA severity [9]. A meta-analysis by Altman et al. concluded that HA injections provide effective pain relief in knee OA and are associated with a more favorable safety profile than nonsteroidal anti-inflammatory drugs (NSAIDs), particularly in older individuals or those with comorbidities [10]. Our findings extend this evidence by showing a continued decline in pain beyond the typical early response phase, suggesting a prolonged clinical benefit. Notably, these outcomes were achieved without adjunctive corticosteroids or systemic analgesics, highlighting the independent therapeutic potential of the HA formulation.

Several aspects of the study design enhance its relevance. The extended 28-week follow-up enabled assessment of both short- and mid-term outcomes. Inclusion of real-world patients—many of whom were refractory to conservative therapy or unsuitable for systemic pharmacologics—supports generalizability. The use of both objective (KL grade, VAS scores) and subjective (patient satisfaction) measures, along with bilateral knee analysis, reflects routine clinical decision-making scenarios.

Radiographic assessments showed stable Kellgren–Lawrence (KL) appearance throughout the study, indicating no acute or unexpected structural changes following intra-articular injection. The inclusion of an early radiographic follow-up at Week 6 was primarily for post-injection safety monitoring and to ensure structural stability rather than for detecting early osteoarthritic changes. However, KL grading has limited sensitivity for early structural changes. Future studies incorporating MRI-based cartilage assessment or synovial biomarkers may provide deeper insights into potential disease-modifying effects of HA therapy.

Safety outcomes were favorable. Among 65 participants, only two treatment-related adverse events (3.1%) were reported—both mild and self-limiting. No systemic reactions or treatment-related serious adverse events (SAEs) occurred. These findings are in line with post-marketing surveillance (PMS) data, where intra-articular HA has been associated with SAE rates under 1% and withdrawal rates due to AEs below 5%. Observational data also support the favorable safety profile of HA injections across multicenter settings and elderly populations [13–15].

Recent evidence from a comprehensive systematic review of post-marketing surveillance studies across anti-osteoarthritis medications further supports these observations. The review reported that PMS data generally corroborate the safety profiles established in phase–3 clinical trials, while emphasizing the importance of robust real-world monitoring frameworks. Notably, intra-articular hyaluronic acid (IA-HA) was among the most frequently assessed interventions (16 PMS studies), with safety signals largely limited to transient local reactions and serious adverse events being uncommon. These findings are consistent with our post-market open-label study, in which no treatment-related SAEs were observed and injection-site reactions were infrequent and mild, supporting the overall tolerability and structural safety of IA-HA over 28 weeks [16].

Mechanistically, HA is thought to exert its effects through both biomechanical and biological mechanisms. Besides restoring viscoelastic properties of the synovial fluid, HA interacts with CD44 and Toll-like receptors (TLR2/4) on synoviocytes and immune cells, reducing pro-inflammatory cytokines (e.g., IL-1β, TNF-α) and matrix metalloproteinases (MMPs) that contribute to cartilage breakdown [17–22]. Preclinical studies also suggest that HA enhances endogenous HA synthesis, reduces oxidative stress, and promotes chondrocyte survival [23, 24]. These mechanisms may collectively account for the observed symptomatic relief and structural stability.

Several limitations of this study warrant consideration. First, the open-label, single-arm design limits the ability to directly compare outcomes with a placebo or active comparator. Nonetheless, given the substantial body of evidence supporting intra-articular HA—and ethical concerns associated with withholding treatment in symptomatic patients—a single-arm design was considered appropriate in this post-market context.

Second, while the sample size was adequate for evaluating overall safety and clinical trends, it may not have been sufficient to detect subtle radiographic changes or to support exploratory analyses using advanced imaging modalities such as MRI. Future studies with larger cohorts and higher-resolution assessment tools are warranted to evaluate potential disease-modifying effects.

Third, although the use of Kellgren–Lawrence (KL) grading provides a widely accepted radiographic standard, it lacks sensitivity to detect early structural changes; incorporation of MRI or biomarker-based evaluations could enhance future investigations.

Lastly, the exclusion of participants using systemic analgesics—while intended to minimize confounding—may limit the generalizability of the findings to broader OA populations requiring pharmacologic symptom control.

Importantly, this single-arm post-market clinical follow-up (PMCF) study was conducted in accordance with international regulatory guidelines for Class III medical devices and offers valuable real-world safety and performance data in a clinically relevant population often underrepresented in randomized controlled trials.

Given that the study population comprised primarily older adults with KL grade II–III knee osteoarthritis who were not suitable candidates for systemic pharmacologic interventions, the findings may be most generalizable to this real-world subgroup. Further studies are warranted to explore the applicability of intra-articular HA in younger patients or those with more advanced disease.

In summary, this study contributes to the growing real-world evidence base supporting intra-articular HA as a safe and effective treatment for knee OA. The combination of sustained pain relief, radiographic stability, and high patient satisfaction over 28 weeks underscores its continued utility in routine clinical practice.

## Supplementary information

Below is the link to the electronic supplementary material.

ESM 1 (PDF 232 KB)

## Data Availability

The de-identified datasets generated and analyzed during the current study are available from the corresponding author on reasonable request.

## References

[1] Primorac D, Molnar V, Rod E, Jelec Z, Cukelj F, Matisic V, Vrdoljak T, Hudetz D, Hajsok H, Boric I (2020) Knee osteoarthritis: a review of pathogenesis and state-of-the-art non-operative therapeutic considerations. Genes (Basel) 11(8):854. 10.3390/genes11080854 PMC7464436 32722615

[2] Wu R, Guo Y, Chen Y, Zhang J (2025) Osteoarthritis burden and inequality from 1990 to 2021: a systematic analysis for the global burden of disease study 2021. Sci Rep 15(1):8305. 10.1038/s41598-025-93124-z PMC11894191 40065123

[3] Bruyere O, Honvo G, Veronese N, Arden NK, Branco J, Curtis EM, Al-Daghri NM, Herrero-Beaumont G, Martel-Pelletier J, Pelletier JP et al (2019) An updated algorithm recommendation for the management of knee osteoarthritis from the European Society for Clinical and Economic Aspects of Osteoporosis, Osteoarthritis and Musculoskeletal Diseases (ESCEO). Semin Arthritis Rheum 49(3):337–50. 10.1016/j.semarthrit.2019.04.008 31126594

[4] Salis Z, Sainsbury A (2024) Association of long-term use of non-steroidal anti-inflammatory drugs with knee osteoarthritis: a prospective multi-cohort study over 4-to-5 years. Sci Rep 14(1):6593. 10.1038/s41598-024-56665-3 PMC10950850 38504099

[5] Domzalski M, Migliore A (2022) A review of the clinical effectiveness and safety of hybrid cooperative complexes in intra-articular viscosupplementation. Rheumatol Ther 9(4):957–974. 10.1007/s40744-022-00450-z PMC9314521 35501596

[6] Giaretta S, Magni A, Migliore A, Natoli S, Puntillo F, Ronconi G, Santoiemma L, Sconza C, Viapiana O, Zanoli G (2024) A review of current approaches to pain management in knee osteoarthritis with a focus on Italian clinical landscape. J Clin Med 13(17):5176. 10.3390/jcm13175176 PMC11396710 39274389

[7] Kogan G, Soltes L, Stern R, Gemeiner P (2007) Hyaluronic acid: a natural biopolymer with a broad range of biomedical and industrial applications. Biotechnol Lett 29(1):17–25. 10.1007/s10529-006-9219-z 17091377

[8] Corvelli M, Che B, Saeui C, Singh A, Elisseeff J (2015) Biodynamic performance of hyaluronic acid versus synovial fluid of the knee in osteoarthritis. Methods 84:90–98. 10.1016/j.ymeth.2015.03.019 PMC4526414 25858258

[9] Altman RD, Manjoo A, Fierlinger A, Niazi F, Nicholls M (2015) The mechanism of action for hyaluronic acid treatment in the osteoarthritic knee: a systematic review. BMC Musculoskelet Disord 16:321. 10.1186/s12891-015-0775-z PMC4621876 26503103

[10] Altman R, Bedi A, Manjoo A, Niazi F, Shaw P, Mease P (2019) Anti-inflammatory effects of intra-articular hyaluronic acid: a systematic review. Cartilage 10(1):43–52. 10.1177/1947603517749919 PMC6376563 29429372

[11] Conrozier T, Raman R, Diracoglu D, Montfort J, Bard H, Baron D, Goncalves B, Richette P, Migliore A, Chevalier X et al (2024) EUROVISCO consensus guidelines for the use of hyaluronic acid viscosupplementation in knee osteoarthritis based on patient characteristics. Cartilage 19476035241271970. 10.1177/19476035241271970 PMC11577334 39564753

[12] Charan J, Biswas T (2013) How to calculate sample size for different study designs in medical research? Indian J Psychol Med 35(2):121–126. 10.4103/0253-7176.116232 PMC3775042 24049221

[13] Uthman I, Raynauld JP, Haraoui B (2003) Intra-articular therapy in osteoarthritis. Postgrad Med J 79(934):449–453. 10.1136/pmj.79.934.449 PMC1742771 12954956

[14] Pavelka K, Horvath R, Hurnakova J, Saracino L, Giordan N, Prochazkova L, Moster E, Dokoupilova E (2021) Clinical effectiveness and safety of intra-articular injection of HYALGO in the management of knee osteoarthritis symptoms: a multicenter prospective study. J Clin Orthop Trauma 19:75–80. 10.1016/j.jcot.2021.05.009 PMC8165427 34099970

[15] Migliore A, Granata M (2008) Intra-articular use of hyaluronic acid in the treatment of osteoarthritis. Clin Interv Aging 3(2):365–369. 10.2147/cia.s778 PMC2546480 18686758

[16] Honvo G, Lengelé L, Alokail M, Al-Daghri N, Reginster JY, Bruyère O (2025) Safety of anti-osteoarthritis medications: a systematic literature review of post-marketing surveillance studies. Drugs 85(4):505–555. 10.1007/s40265-025-02162-4 40095377

[17] Sherman SL, Gudeman AS, Kelly JDt, Dimeff RJ, Farr J (2025) Mechanisms of action of intra-articular hyaluronic acid injections for knee osteoarthritis: a targeted review of the literature. Am J Sports Med 53(11):3635465241302820. 10.1177/03635465241302820 PMC12381393 40108507

[18] Gallagher B, Tjoumakaris FP, Harwood MI, Good RP, Ciccotti MG, Freedman KB (2015) Chondroprotection and the prevention of osteoarthritis progression of the knee: a systematic review of treatment agents. Am J Sports Med 43(3):734–744. 10.1177/0363546514533777 24866892

[19] Bucci J, Chen X, LaValley M, Nevitt M, Torner J, Lewis CE, Felson DT (2022) Progression of knee osteoarthritis with use of intraarticular glucocorticoids versus hyaluronic acid. Arthritis Rheumatol 74(2):223–226. 10.1002/art.42031 PMC8795477 34807518

[20] Frizziero L, Govoni E, Bacchini P (1998) Intra-articular hyaluronic acid in the treatment of osteoarthritis of the knee: clinical and morphological study. Clin Exp Rheumatol 16(4):441–449 9706425

[21] Campo GM, Avenoso A, D’Ascola A, Scuruchi M, Prestipino V, Calatroni A, Campo S (2012) Hyaluronan in part mediates IL-1beta-induced inflammation in mouse chondrocytes by up-regulating CD44 receptors. Gene 494(1):24–35. 10.1016/j.gene.2011.11.064 22192912

[22] Wu PT, Kuo LC, Su FC, Chen SY, Hsu TI, Li CY, Tsai KJ, Jou IM (2017) High-molecular-weight hyaluronic acid attenuated matrix metalloproteinase-1 and -3 expression via CD44 in tendinopathy. Sci Rep 7:40840. 10.1038/srep40840 PMC5238506 28091588

[23] Gupta RC, Lall R, Srivastava A, Sinha A (2019) Hyaluronic acid: molecular mechanisms and therapeutic trajectory. Front Vet Sci 6:192. 10.3389/fvets.2019.00192 PMC6603175 31294035

[24] Herrero-Beaumont G, Marco-Bonilla M, Migliore A, Lopez-Reyes A, Irigaray-Moreno A, Mediero A, Largo R (2025) Impact of intra-articular high-concentration hyaluronic acid administration on the innate immune response in experimental knee osteoarthritis. BMC Musculoskelet Disord 26(1):778. 10.1186/s12891-025-09061-5 PMC12341135 40797305

